# A Diagnostic Model for Kawasaki Disease Based on Immune Cell Characterization From Blood Samples

**DOI:** 10.3389/fped.2021.769937

**Published:** 2022-01-05

**Authors:** Shangming Du, Ulrich Mansmann, Benjamin P. Geisler, Yingxia Li, Roman Hornung

**Affiliations:** ^1^Institute for Medical Informatics, Biometry and Epidemiology, University of Munich, Munich, Germany; ^2^Pettenkofer School of Public Health, Munich, Germany

**Keywords:** Kawasaki disease, diagnosis, biomarker, CIBERSORT, immune cell, LASSO model

## Abstract

**Background:** Kawasaki disease (KD) is the leading cause of acquired heart disease in children. However, distinguishing KD from febrile infections early in the disease course remains difficult. Our goal was to estimate the immune cell composition in KD patients and febrile controls (FC), and to develop a tool for KD diagnosis.

**Methods:** We used a machine-learning algorithm, CIBERSORT, to estimate the proportions of 22 immune cell types based on blood samples from children with KD and FC. Using these immune cell compositions, a diagnostic score for predicting KD was then constructed based on LASSO regression for binary outcomes.

**Results:** In the training set (*n* = 496), a model was fit which consisted of eight types of immune cells. The area under the curve (AUC) values for diagnosing KD in a held-out test set (*n* = 212) and an external validation set (*n* = 36) were 0.80 and 0.77, respectively. The most common cell types in KD blood samples were monocytes, neutrophils, CD4^+^-naïve and CD8^+^ T cells, and M0 macrophages. The diagnostic score was highly correlated to genes that had been previously reported as associated with KD, such as interleukins and chemokine receptors, and enriched in reported pathways, such as IL-6/JAK/STAT3 and TNFα signaling pathways.

**Conclusion:** Altogether, the diagnostic score for predicting KD could potentially serve as a biomarker. Prospective studies could evaluate how incorporating the diagnostic score into a clinical algorithm would improve diagnostic accuracy further.

## Introduction

Kawasaki disease (KD) is an acute febrile inflammation of infants and children with an unknown etiology and is the leading cause of acquired heart disease for children in developed countries (1–3). Some of its complications such as coronary aneurysms, thrombotic occlusion, and myocardial infarction may be fatal (4). KD is a clinical diagnosis and no specific diagnostic test exists at present. It can sometimes be difficult to distinguish KD from other febrile illnesses, including from infectious etiologies, which may have the same clinical features or occur concomitantly (5), leading to sometimes delayed treatment and complications (6, 7).

Several biomarkers for KD have been identified, but they either have a low specificity or have not yet been validated in a sufficiently large dataset (8–13). As KD is an inflammatory disease, there are some immunological biomarkers that show promise (8). However, since they are based on either flow cytometry or immunohistochemical staining, these biomarkers remain challenging to standardize and implement into routine clinical practice. Because of these limitations, researchers have begun to look for new diagnostic approaches, including approaches based on high-throughput screening (10, 13, 14). However, the role of the composition of the whole blood immune cells in these approaches remains unclear.

As a novel algorithm based on transcriptomic profiling, Cell-type Identification by Estimating Relative Subsets of RNA Transcripts (CIBERSORT) has been shown to be highly accurate in identifying immune cell types. It remains unknown whether CIBERSORT could be used in distinguishing between KD and FC. Therefore, our objective was to characterize the immune cell composition in KD patients through CIBERSORT and contrast it to those of infectious febrile controls (FCs) to develop a diagnostic score as a potential biomarker for diagnosing KD.

## Materials and Methods

### Data Sources and Pre-processing

Data obtained from microarray samples generated using Illumina® and Stanford Functional Genomics Facility were obtained from the Gene Expression Omnibus (GEO, https://www.ncbi.nlm.nih.gov/geo). Therefore, there was no requirement for institutional review board approval. All KD blood samples were collected from children with KD diagnosed based on the American Heart Association criteria (4), and all FC blood samples were collected from children with a fever and then restricted to cases of definite bacterial, definite viral, and “uncertain” infections (4, 11, 13, 14). Samples from healthy controls were only used in batch-effect removal but were excluded from model building and validation (see below). To construct the discovery cohort, we incorporated four GEO datasets (GSE73461, GSE73462, GSE73463, and GSE68004), including 301 KD, 408 FC and 208 healthy control (HC) samples. Dataset GSE15297, which contained 23 KD and 18 FC samples, was used as the validation cohort.

Raw data from Illumina® microarray were processed using the “lumi” R package (15). Probes were filtered to include only those with a detection *p*-value smaller than 0.01 in at least one sample in every group, and the largest level of probe was selected for a gene detected by multiple probes. Sample outliers and batch effects were identified and assessed using principal component analysis. One sample from GSE73461 was identified as an outlier and excluded (Supplementary Figures 1A,B). The “ComBat” function in the “SVA” R package (16) was applied to correct for batch effects between the datasets. Healthy controls were used in batch-effect removal but were excluded from model building.

Supplementary Table 1 gives an overview of the data. Corresponding authors were contacted for further information where necessary.

### Estimation of Immune Cell Composition

To estimate the proportions of immune cells, we applied CIBERSORT (17), a deconvolution algorithm that can enumerate cell type composition in gene expression data and produce a *p*-value for the deconvolution for each sample using a Monte Carlo approach. This algorithm was run with the default LM22 signature matrix downloaded from the CIBERSORT portal and 1,000 permutations using the “CIBERSORT” R package. Only samples with CIBERSORT *p*-value smaller than 0.05 were included.

### Functional and Pathway Enrichment Analysis

We also investigated enriched biological processes and pathways between high- and low-diagnostic score groups by running a gene set enrichment analysis (GSEA) (18) using the “clusterProfiler” and “DOSE” packages (19) in R. Reference gene sets were downloaded from the MSigDB database of the Broad Institute (20) including “h.all.v7.4,” “c2.cp.kegg,” and “c2.cp.biocarta,” which were applied to quantity activities of the corresponding pathways. Target terms were identified with the strict cut-off 0.05 in the enrichment *p*-values based on 1,000 permutations. The *p*-values were adjusted for multiple testing using the Benjamini-Hochberg procedure.

### Unsupervised Clustering

In order to classify all febrile patients into different molecular subtypes, we performed unsupervised clustering using the “ConsensusClusterPlus” R package (21). The consensus clustering was based on the K-means algorithm, with 1,000 iterations using 80% of the samples selected randomly. The optimal cluster number was determined using the consensus cumulative distribution function.

### Statistical Analysis

Samples in GSE73461, GSE73462, GSE73463, and GSE68004 were combined into the discovery cohort and subsequently randomly separated into training and test set (7:3) for identifying and evaluating the models. The test set will be referred to as the “held-out test set” in the following. For external validation, the GSE15297 dataset was used.

The least absolute shrinkage and selection operator (LASSO) algorithm for binary outcomes implemented in the “glmnet” R package (22) was applied to identify the most important diagnostic immune cells using the training set. The optimal value of the penalty regularization parameter λ was determined using 10-fold cross-validation and the 1-SE criterion. Subsequently, the identified variables were used as covariates in logistic regression to obtain the diagnostic prediction model. Note that the LASSO already delivers a fully specified model, which is why using logistic regression as a second step would not have been necessary to arrive at a diagnostic prediction model. However, for the case of continuous outcomes this two-step procedure has been shown to deliver less biased coefficient estimates compared to the LASSO (23). Prediction performance was evaluated using receiver-operating characteristic (ROC) curves. Optimal cut-off points were determined by maximizing Youden's index using the “OptimalCutpoints” R-package (24). For continuous variables, group comparisons were performed using the Wilcoxon test because there were indications of violations of the normality assumption for these data. For categorical variables, Fisher's exact test was used. Spearman's rank correlation coefficient was used to analyze correlations between the diagnostic score and the expression levels of genes. All statistical tests were two-sided. *P-*values and false discovery rates smaller than 0.05 were considered statistically significant.

## Results

### Patient Characteristics

**Figure 1:**
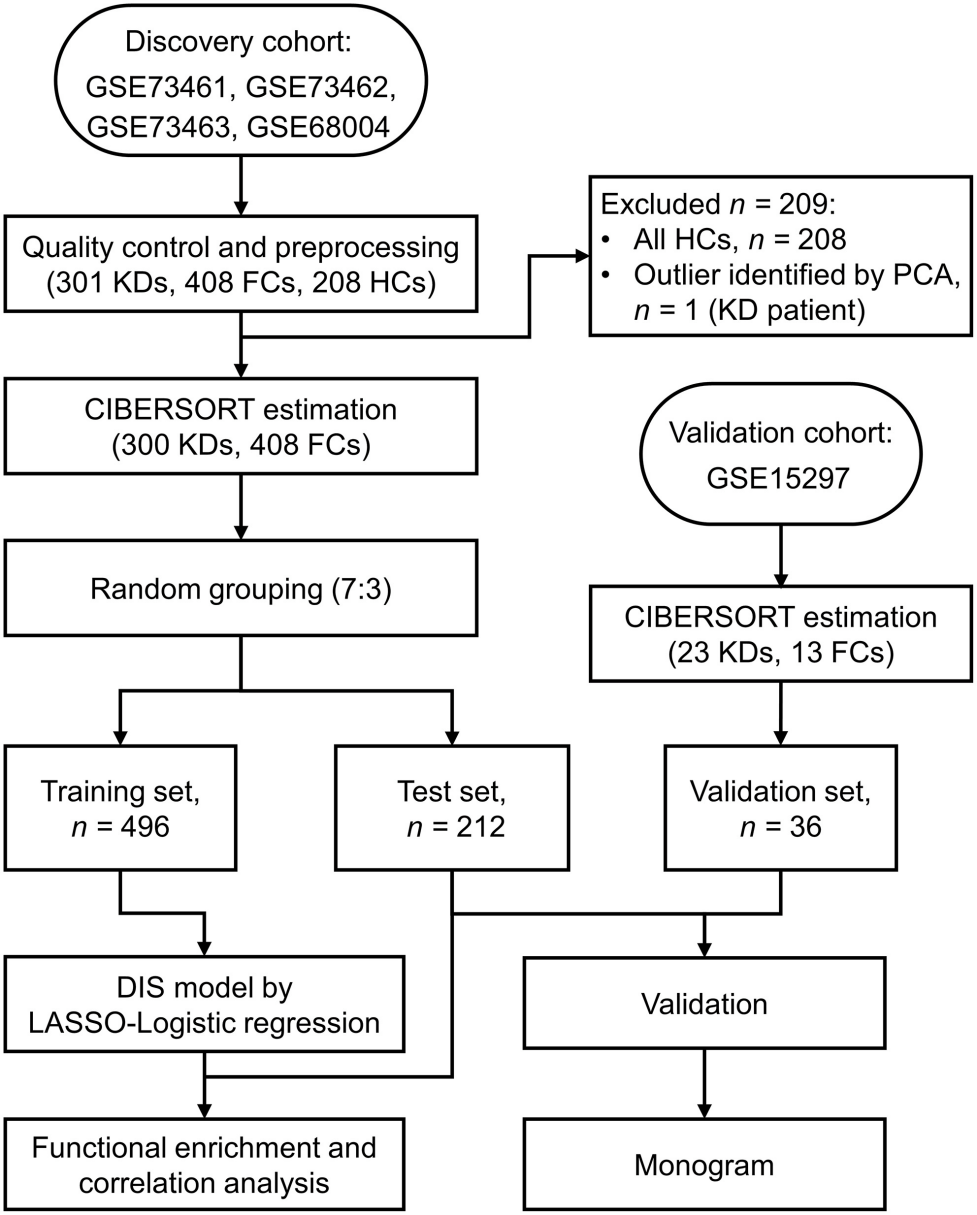
Flow chart of the study design. KD, Kawasaki disease; FC, febrile control; HC, healthy control; PCA, Principal component analysis; CIBERSORT, cell-type identification by estimating relative subsets of RNA transcripts; LASSO, least absolute shrinkage and selection operator.

Figure 1 provides a detailed overview of the workflow. After applying the filter criteria, 708 children (417 males and 291 females) were included in the discovery cohort, containing 300 KDs and 408 FCs (92 definite bacterial, 141 definite viral, and 175 uncertain infections), which was subsequently randomly divided into a training set and a held-out test set. For external validation, we used the GSE15297 dataset, including 23 KDs, eight definite bacterial infections and five definite viral infections. Baseline demographic and clinical characteristics of the patients are listed in Table 1.

**Table 1:** Clinical characteristics in the different datasets.

	Discovery^*^			**Validation**
	**Entire set**	**Training set**	**Test set**	**Validation set**
Number of patients	708	496	212	36
Age, months	27.0 (9.0–59.0)	27.0 (9.0–54.0)	29.0 (8.0–66.0)	NA
**Sex**				
Male	417 (58.9)	291 (58.7)	126 (59.4)	23 (63.9)
Female	291 (41.1)	205 (41.3)	86 (40.6)	13 (36.1)
**Outcome**				
Kawasaki disease	300 (42.4)	210 (42.3)	90 (42.5)	23 (63.9)
Febrile controls	408 (57.6)	286 (57.7)	122 (57.5)	13 (36.1)
**Pathogens in febrile controls**				
Definite bacterial	92 (22.5)	70 (24.5)	22 (18.0)	8 (61.5)
Definite viral	141 (34.6)	95 (33.2)	46 (37.7)	5 (38.5)
Uncertain	175 (42.9)	121 (42.3)	54 (44.3)	0

*Data are presented as median (IQR), or n (%); NA, not applicable*.

* *There were no significant differences between children in Training and Test sets*.

### Composition of Immune Cells in KD and FC Blood Samples

**Figure 2:**
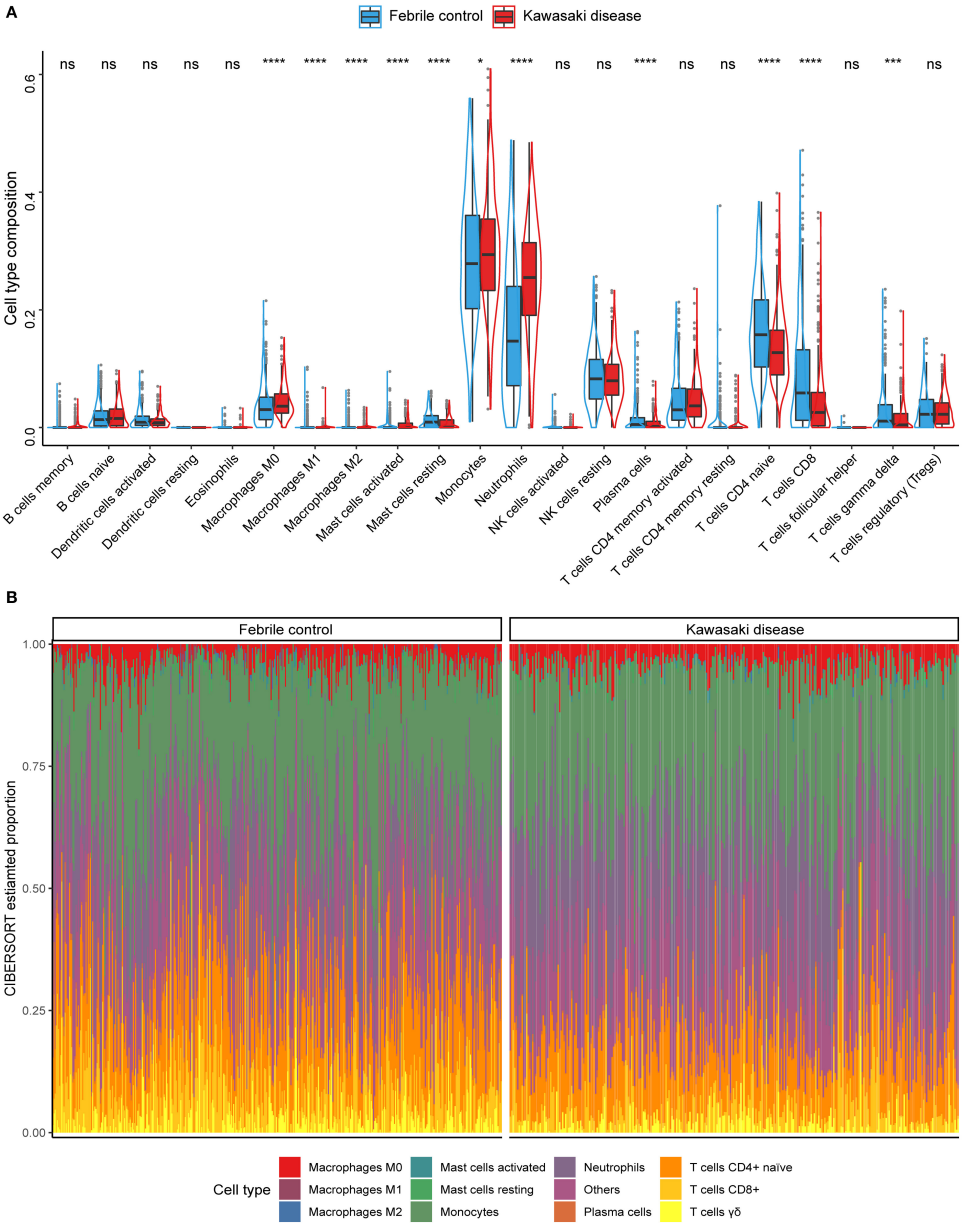
Summary of the immune cell types inferred using the CIBERSORT algorithm. **(A)** The compositions of immune cells of the KDs (red) vs. the FCs (blue) in the discovery cohort. Wilcoxon test: *p < 0.05; ***p < 0.001; ****p < 0.0001; ns, not significant. **(B)** Stacked bar plots indicating the estimated proportions of 11 immune cells with significant difference (KD vs. FC) and other cell types for each sample in the discovery cohort.

We used the CIBERSORT algorithm to analyze the composition of immune cells in each individual sample. The proportions of activated mast cells (*p* = 0.035), M0 macrophages (*p* < 0.001), monocytes (*p* = 0.035), and neutrophils (*p* < 0.001) were significantly higher in KD samples than in FC samples. In contrast, the fractions of M1 and M2 macrophages, activated mast cells, plasma cells, CD4^+^-naïve and CD8^+^ T cells, and γδ T cells (all *p* < 0.001) were lower in KD samples (Figure 2A). In general, the five most common cell types in KD blood samples were monocytes, neutrophils, CD4^+^-naïve, CD8^+^ T cells, and M0 macrophages, accounting for over three quarters of all cell types. Furthermore, we also investigated the ratio of CD4^+^ to CD8^+^ T cells, since it was reported as a potential distinction between the KD and the infectious febrile children (25, 26). Compared to the FC group, KD patients had significantly higher ratios of CD4^+^/CD8^+^ T cells (*p* < 0.001; Supplementary Table 2). Figure 2B further illustrates that the immune cell composition landscape significantly differed between KD and FC samples. In addition, we compared them to the healthy control group (HCs). For most cell types, higher fractions seen in the comparison of HC and KD were also seen in the comparison of FC and KD, and vice versa. An exception was the fraction of plasma cells, which was lower in the comparison of HC and KD, but higher in the comparison of FC and KD (Supplementary Figure 2).

### Derivation and Validation of the Diagnostic Score

**Figure 3:**
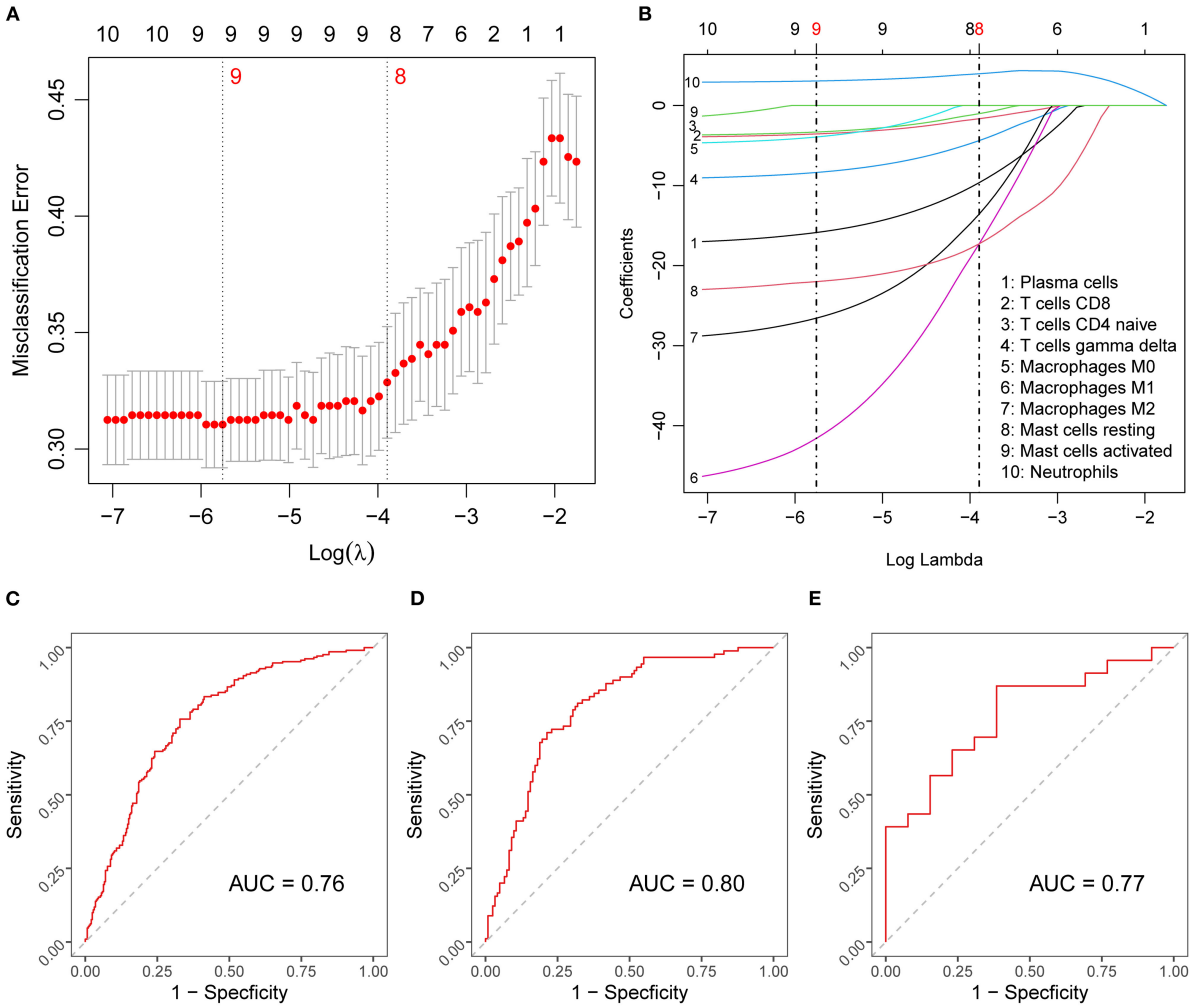
Diagnostic score model construction and validation. **(A)** Results of the 10-fold cross-validation for tuning parameter selection in the LASSO model. The error bars show necessarily biased (27) estimates of the standard errors of the cross-validation estimates. **(B)** LASSO coefficient paths of the fractions of the candidate immune cells. Vertical lines are drawn at the values optimal according to the minimum (left line) and the 1-SE (right line) criterion, and the red numbers represent the numbers of selected variables according to the respective criteria. **(C–E)** Diagnostic score model performance assessed by ROC curves in the training **(C)**, test **(D)**, and validation **(E)** set. LASSO, least absolute shrinkage and selection operator; SE, standard error; ROC, receiver-operating characteristic; AUC, area under ROC curve.

To begin, we extracted the proportions of immune cells with highly significant differences (*p* < 0.01) to construct our diagnostic model. Using LASSO regression for binary outcomes, we narrowed the candidate cell types down to eight variables, where the penalty regularization parameter λ was determined using 10-fold cross-validation and the 1-SE criterion (Figures 3A,B). These eight variables were subsequently used in logistic regression to obtain the diagnostic prediction model. The scores of the selected cell types for this model were continuous variables. The estimated coefficients of the model can be found in Supplementary Table 3. ROC curves were used to evaluate the overall performance of the model. The area under the curve (AUC) was 0.76 in the training set (Figure 3C) and 0.80 in the held-out test set (Figure 3D).

To investigate the diagnostic potential of the diagnostic score in external prediction settings, we evaluated its performance on the independent dataset GSE15297, which resulted in an AUC of 0.77 (Figure 3E), that is, a value very similar to that obtained for the held-out test set.

### Model Performance Diagnosis and Association With KD Related Genes

**Figure 4:**
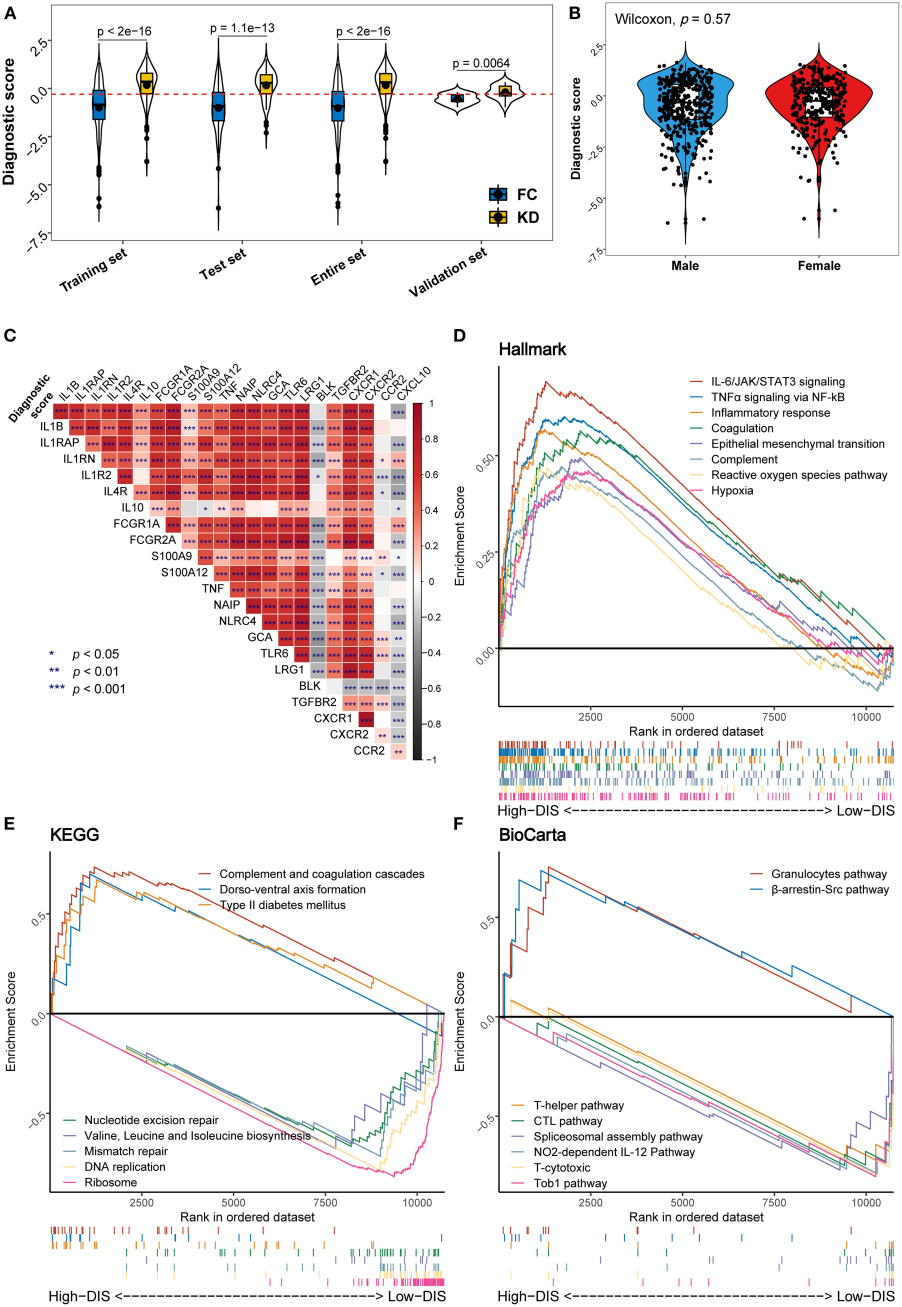
Distribution of the diagnostic score and biological functional analysis. **(A)** Distribution of the diagnostic score in the different studied datasets. In each set, the box plot inside the violin plot represents the median value and the interquartile range of the diagnostic score, and the black point indicates the mean value. The horizontal dashed red line represents the cut-off used to discriminate patients as having KD (above the line) or infectious diseases (below the line). **(B)** Comparison of diagnostic scores between male (blue) and female (red) patients. **(C)** Correlation heat map between the diagnostic score and the expression levels of genes previously reported to be associated with KD. **(D–F)** Gene set enrichment analysis (GSEA) displays biological processes and pathways using the gene sets of “h.all.v7.4” **(D)**, “c2.cp.kegg” **(E)**, and “c2.cp.biocarta” **(F)**. In each figure, the top eight results ordered by enrichment score are shown in different colors.

Using violin plots, we compared the disease-specific distributions of the diagnostic score in the training, test, and validation data to evaluate the model's ability to distinguish KD from other febrile conditions due to infections (Figure 4A). Here, we also derived an optimal cut-off point of −0.295, which was determined by maximizing Youden's index. The plot shows that the diagnostic score was higher in KD samples in each of the datasets. The distributions of the diagnostic score are very similar between the training and test data but differ in the validation data. The values of the diagnostic score had different distributions between the discovery cohort and the validation cohort. We think this difference is due to the different microarray platforms underlying the discovery and validation datasets. The different gene-probe compositions and different preprocessing pipelines underlying the validation dataset might lead to different data input in the CIBERSORT algorithm. However, the diagnostic score remains valid because, for every dataset, the diagnostic score values of KDs are significantly higher than those of FCs and the cut-off point separates the two diseases quite well. In addition, we investigated the association of the diagnostic score with gender and found that the diagnostic score values did not vary significantly between male and female children (*p* = 0.57) (Figure 4B). Correlation analysis showed that the diagnostic score correlated significantly positively with the expression levels of most genes previously reported to be related to KD (28, 29) (Figure 4C). To further provide a practical quantitative tool that allows for application of our diagnostic model in clinical practice, we constructed a nomogram incorporating these immune cell predictors (Supplementary Figure 3A). Lastly, we calculated calibration curves and decisions curves separately for the training, test, and validation data. The calibration curves revealed a good correspondence between predicted and actual outcome values and the decision curves suggested that the net benefit of treatment based on the decision of the model is larger than that of treating all or no patients for most possible risk thresholds (Supplementary Figures 3B–G).

### Biological Functions Associated With the Diagnostic Score

To assess biological plausibility, we performed GSEA, focusing on three reference gene sets. Downloaded from the MSigDB database, these gene sets consisted of well-defined biological processes (Hallmark: “h.all.v7.4”) and two canonical pathways (KEGG: “c2.cp.kegg”; BioCarta: “c2.cp.biocarta”). In this analysis, we used the previously mentioned cut-off point for the diagnostic score. Hallmark inflammatory pathways including IL-6/JAK/STAT3 signaling, TNFα signaling, inflammatory response, the complement, and reactive oxygen species pathways were enriched in the high-diagnostic score group compared to the low-diagnostic score group (Figure 4D). Similarly, KEGG pathway analysis suggested an enrichment for the complement and coagulation cascades. On the other hand, a low diagnostic score was associated with gene replication and repair, as well as protein biosynthesis processes (Figure 4E). Likewise, BioCarta pathways analysis indicated that immune-related pathways (granulocytes and β-arrestin/Src pathways) were relatively over-expressed in the high-diagnostic score group, whereas acquired immune response pathways appeared to be mainly enriched in the low-diagnostic score group (Figure 4F).

### Molecular Subtypes of Febrile Children

**Figure 5:**
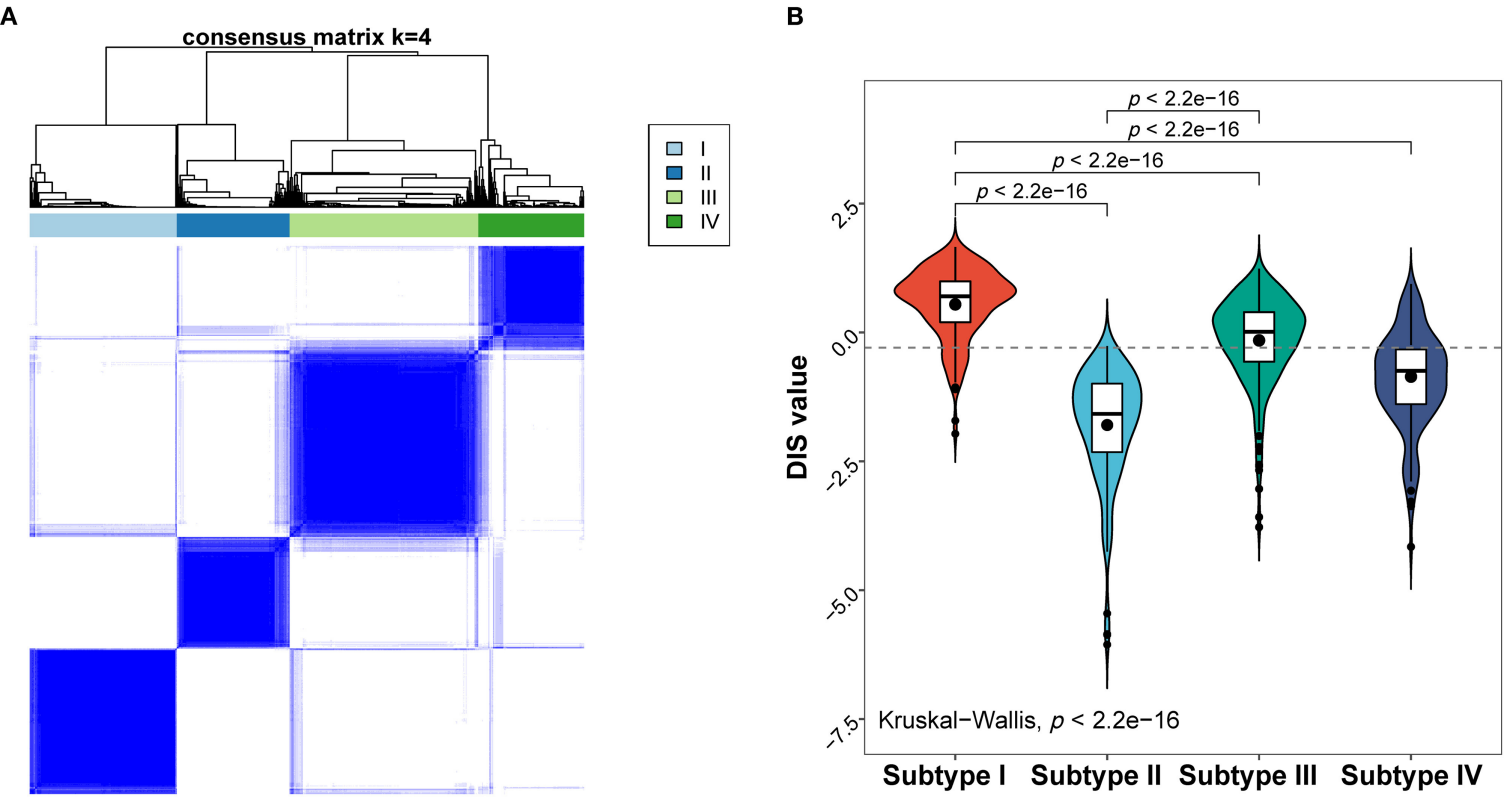
Molecular subtypes of the febrile condition cases obtained based on their gene expression profiles. **(A)** Heat map of the consensus matrix with four clusters obtained using unsupervised clustering. **(B)** Distribution of the diagnostic score in different molecular subtypes; *p*-values are only shown for comparisons with statistically significant differences.

To identify possible patterns of molecular subtypes of all febrile condition cases, we carried out unsupervised consensus clustering on the entire discovery cohort. Notably, four potential molecular subtypes were found using the consensus cumulative distribution function (Figure 5A; Supplementary Figures 4A,B). In addition, assessment of the distribution of the diagnostic score in the identified subtypes (Figure 5B) showed that molecular subtypes would be associated with the diagnostic score. Moreover, Subtype I was considered a high-risk subtype of KD, whereas Subtype II seemed to have received risk score values similar to those associated with infectious illnesses (Figure 5B; Supplementary Table 4).

## Discussion

In this study, we developed a diagnostic score for KD, consisting of the fractions of eight immune cells, selected based on a combination of machine learning techniques. The diagnostic score was able to differentiate well between KD and FC patients (AUC = 0.8). We also elucidated the roles of the immune-related genes and pathways in KD, such as interleukins and chemokine receptors, as well as IL-6/JAK/STAT3 and TNFα signaling pathways and identified molecular subtypes of febrile children using unsupervised learning. Nevertheless, the practicability and generalizability of this model might be limited because all analyzed data were obtained from a public database, which is why our results should be further validated – ideally prospectively.

Effects of the immune microenvironment on the pathogenesis of KD have been well-reported in many studies, and most of them underscore the importance of the innate immune system in the acute phase of KD. Particularly, levels of monocytes/macrophages and neutrophils were markedly upregulated in children with KD (25, 30, 31). Our results were also in agreement with these reports from high-throughput studies (30, 31). In our diagnostic score, the proportion of macrophages and neutrophils weighed heavy. Due to the lack of experimental validation, we conducted computational analysis for biological functions, which were necessarily speculative. Our results showed that a high diagnostic score might also be associated with innate immune-related genes and signaling pathways such as S100 proteins, certain chemokines, IL-6/JAK/STAT3 signaling, and TNFα signaling pathways. Furthermore, the imbalance in T cell subsets was reported as explanatory for the differences between KD and FC. Furukawa et al. (26) and Ding et al. (25) suggested that KD patients have a significantly higher ratio of CD4^+^/CD8^+^ T cells compared to the FC group. Our results were consistent with this finding to a certain extent. Nevertheless, Xu et al.'s findings (32) suggest that B cell subsets have a role in KD. In contrast, we found that the presence of plasma cells had a negative influence on the probability for KD in our diagnostic score, and there seemed to be no significant difference between the proportions of memory or naïve B cells between the two groups. This may be the case because Xu et al. compared KD cases to healthy controls instead of FCs, and also because certain B cell subsets can indicate the presence of an infection. Genes in the same disease subtypes have similar expression patterns. Therefore, we also applied unsupervised consensus clustering to identify molecular subtypes with similar gene expression patterns. Among the identified subtypes, Subtype I and Subtype II were mainly predicted to be KD and FC, respectively, providing genetic evidence for the validity of our model.

KD is currently diagnosed based on clinical criteria plus, in some instances, additional non-specific laboratory testing (4, 7, 10). However, KD can sometimes be confused with other febrile illnesses, including infections, because of their mutual clinical manifestations, and this may result in delayed treatment from which complications may arise (6, 7, 33). Conversely, given the gravity of the diagnosis, overtreatments with intravenous immunoglobulin or other immunosuppressants may occur in incorrectly diagnosed cases (13). A biomarker that accurately distinguishes KD from infectious febrile disease would therefore be a major advancement, reducing inappropriate treatment and allowing for early intravenous immunoglobulin therapy in true cases. We developed the present diagnostic model as a contribution to such a biomarker, applying machine-learning methods to high-throughput data rather than clinical features. ROC curve analysis suggested that our diagnostic model performs has also an acceptable performance in external prediction settings. However, prospective studies would be necessary to compare the performance of the diagnostic score model with that of the established clinical algorithm for KD. Rather than comparing the current clinical practice with the present model head-to-head, one could develop an integrated model with both clinical and CIBERSORT features which may potentially offer better test characteristics than either by itself. The clinical algorithm, at present, only suggests measuring C-reactive protein and erythrocyte sedimentation rate in cases with an intermediate likelihood of KD (4). This new model could have a significantly better diagnostic performance, particularly for patients with only two or three of the clinical criteria present.

As an immunological disease, KD may be identified using immunological methods. However, flow cytometry or immunohistochemistry seem to be unsuitable for routine use (8). High-throughput methods may be well-suited to characterize the “immune landscape” for KD vs. FC classification. Although some KD biomarkers based on transcriptomic profiling do exist (10, 13, 14), they did not leverage the power of profiling the immune cell types presented. As a combination of high-throughput transcriptomic profiling with “immune landscape” estimation, CIBERSORT seems to be a possible solution to this dilemma.

We acknowledge some limitations of this study. First, a diagnostic score was developed from publicly available datasets, where it was difficult to obtain all demographic and clinical information for each patient. However, the variability of gene expression patterns may be accompanied by the diversity of demographic features such as ethnicity. Second, combining data from different microarray datasets leads to batch-effect affected data. However, principal component analysis suggested that most of the present batch effects have been remedied by employing the “ComBat” algorithm. Third, bias may be associated with the fact that the time since disease onset varied between patients. Unfortunately, the information on the time since disease onset was not complete in the used datasets from GEO database. In future efforts, “days of illness” should be an important factor in data collection. Fourth, bias may have been introduced, because some KD patients may have been mislabeled as not having KD or vice versa. Finally, in view of the recent pandemic of pediatric COVID-19 and the related sever syndrome (multisystem inflammatory syndrome in children, MIS-C), missed or delayed diagnosis of KD is quickly attracting concern (34–36). Thus, in future analyses it would be interesting to apply the analysis flow considered in this paper to the diagnosis of MIS-C and other diseases similar to KD. In the current work, it was not possible to compare the diagnostic score with these diseases due to the lack of sufficient gene expression data on them. However, in the near future, we plan to integrate cohorts on these diseases to improve our model and expand its scope of application.

## Conclusion

In summary, using CIBERSORT cell type compositions, we developed a diagnostic score model which has the potential to serve as a biomarker for early diagnosis of KD. However, prospective studies are necessary to validate the diagnostic score further and ideally incorporate it into a new clinical algorithm to more accurately diagnose or rule out KD.

## Data Availability Statement

Publicly available datasets were analyzed in this study. This data can be found at: Gene Expression Omnibus (GEO) data portal (https://www.ncbi.nlm.nih.gov/geo/; Accession Numbers: GSE73461, GSE73462, GSE73463, GSE68004, and GSE15297).

## Author Contributions

SD designed the project, performed the analysis, and contributed to manuscript writing. UM coordinated the project and contributed to the manuscript writing. BG gave medical input and contributed to manuscript writing. YL contributed to the statistical analysis. RH supervised the statistical analysis, contributed to the bioinformatics analysis, and to manuscript writing. All authors contributed to the article and approved the submitted version.

## Funding

This work was supported by the China Scholarship Council (CSC, No. 201706380046).

## Conflict of Interest

The authors declare that the research was conducted in the absence of any commercial or financial relationships that could be construed as a potential conflict of interest.

## Publisher's Note

All claims expressed in this article are solely those of the authors and do not necessarily represent those of their affiliated organizations, or those of the publisher, the editors and the reviewers. Any product that may be evaluated in this article, or claim that may be made by its manufacturer, is not guaranteed or endorsed by the publisher.
