# Supplementary material for: A Diagnostic Model for Kawasaki Disease Based on Immune Cell Characterization From Blood Samples
Source: Front Pediatr. 2022 Jan 5;9:769937. doi: 10.3389/fped.2021.769937 (PMC8767645; doi:10.3389/fped.2021.769937)
Supplement: Supplementary file 5 [file Data_Sheet_1.docx]

# **Supplementary material legends**

**Figure S1** Principal component analysis using the discovery cohort. Merged dataset before (**A**) and after (**B**) batch-effect removal, respectively.

**Figure S2.** The compositions of immune cells of the HCs (yellow) vs. the FCs (blue) vs.the KDs (red) in the discovery cohort. Wilcoxon test: *, *p* < 0.05; **, *p* < 0.01; ***, *p* < 0.001; ****, *p* < 0.0001; ns, not significant.

**Figure S3** Nomogram construction and validation. (**A**) Nomogram for predicting the risk for KD obtained using the training set. (**B-D**) Calibration curve of the nomogram to study the concordance between the predicted and the actual outcomes in training (**B**), test (**C**) and external validation set (**D**). Perfect predictions would lie on the 45-degree gray line, and the apparent and bias-corrected (by bootstrapping, B=1000 repetitions) performance lines are shown in red and blue. (**E-G**) Decision curve analyses of the nomogram in training (**E**), test (**F**) and external validation set (**G**).

**Figure S4** Diagnostic plots for choosing the optimal number of clusters in the consensus clustering. (**A**) Curve of the Consensus Cumulative Distribution Function (CDF) for different numbers of clusters. (**B**) Curve graph of the delta area. K is the number of selected clusters. The optimal number of clusters is four. For details on consensus clustering see Wilkerson *et al.*^21^

**Table S1** Summary of the datasets.

**Table S2** Comparison of the percentages of the T cell subsets between the KD and the FC group.

**Table S3** Estimated coefficients of the diagnostic prediction model for Kawasaki disease diagnosis.

**Table S4** Relationship between the diagnosis and the identified molecular subtypes.
