# Supplementary figures and images for: A Diagnostic Model for Kawasaki Disease Based on Immune Cell Characterization From Blood Samples
Source: Front Pediatr. 2022 Jan 5;9:769937. doi: 10.3389/fped.2021.769937 (PMC8767645; doi:10.3389/fped.2021.769937)

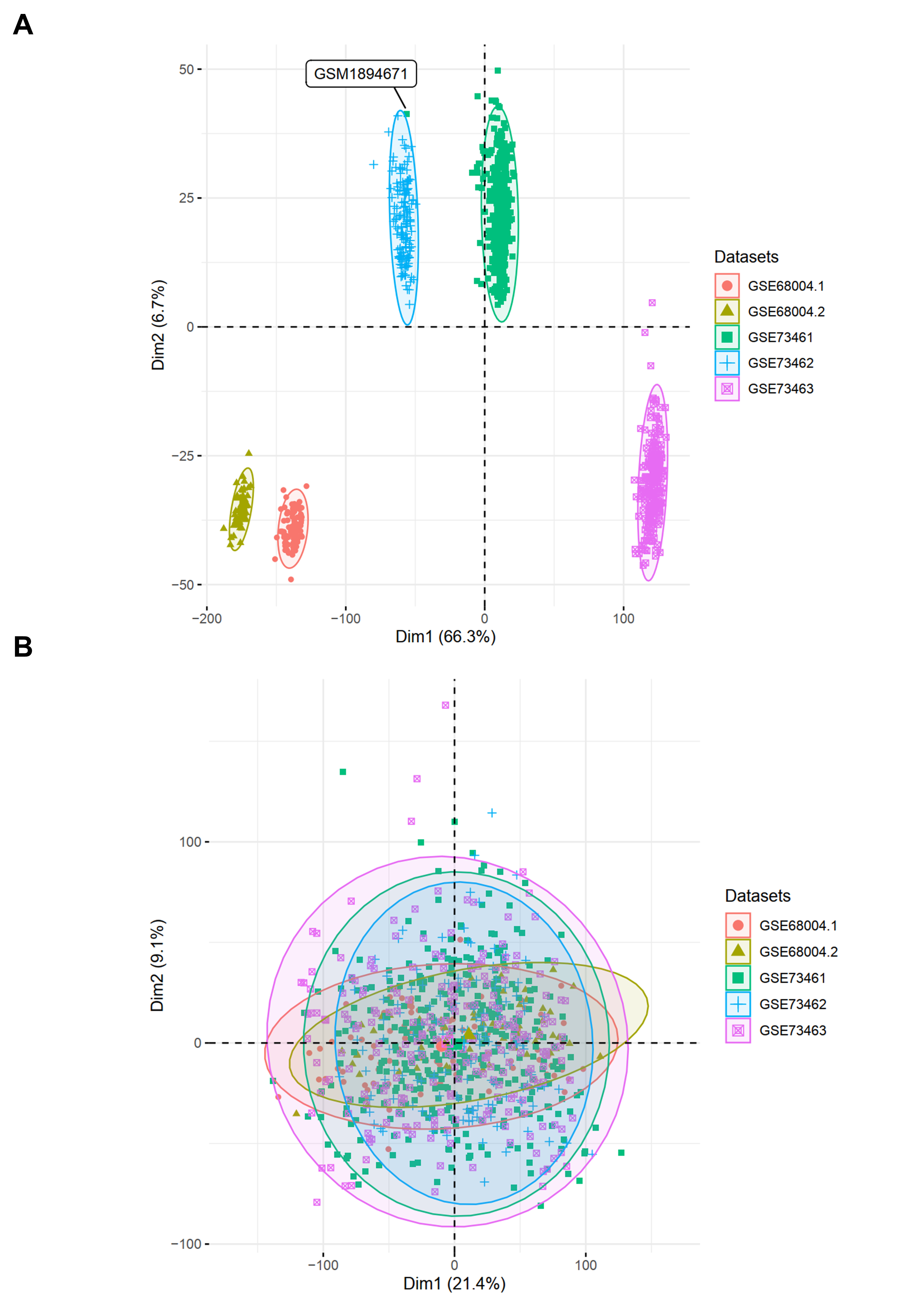

Supplement: Supplementary file 6 [file Image_1.TIF]

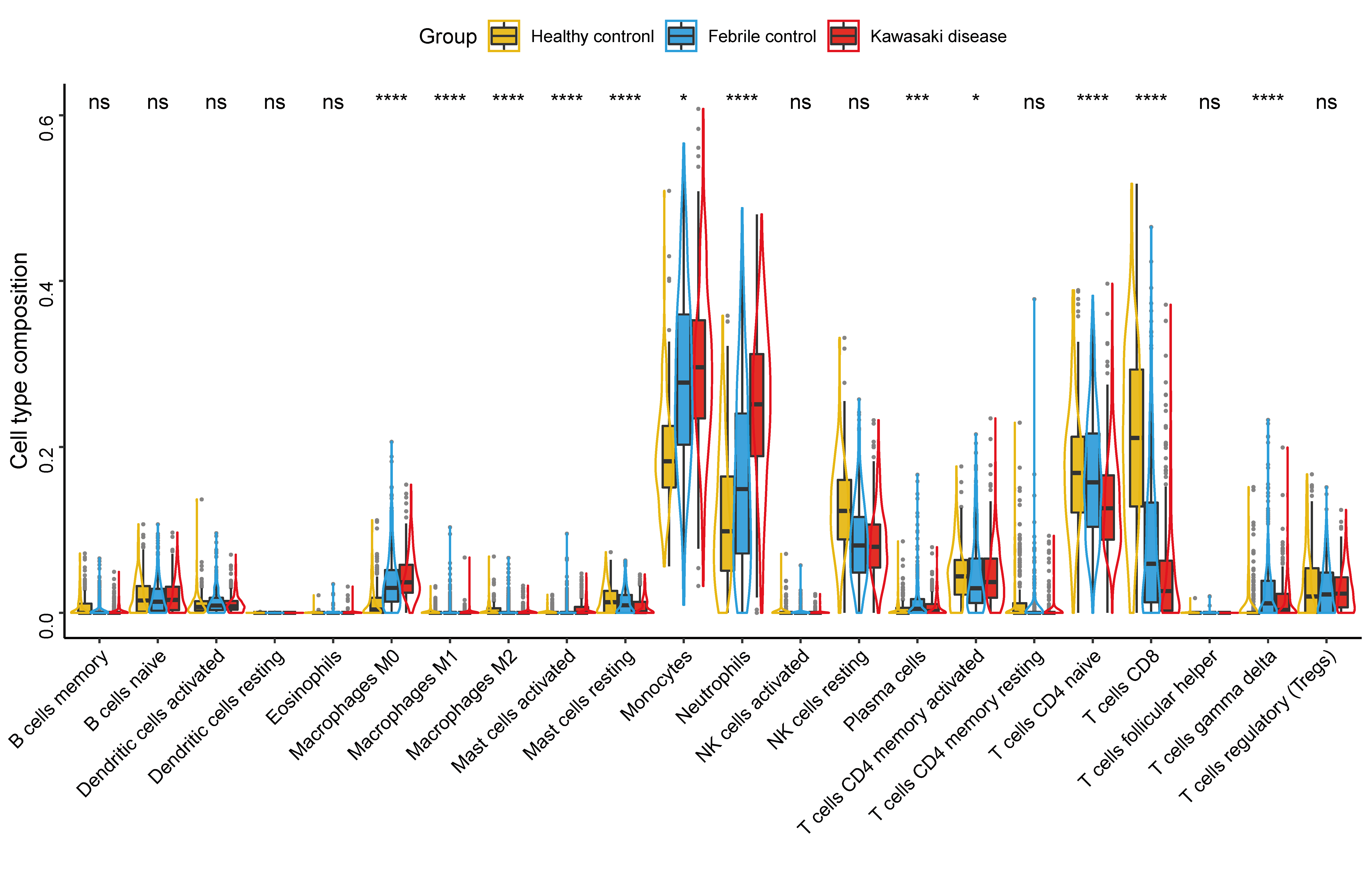

Supplement: Supplementary file 7 [file Image_2.TIF]

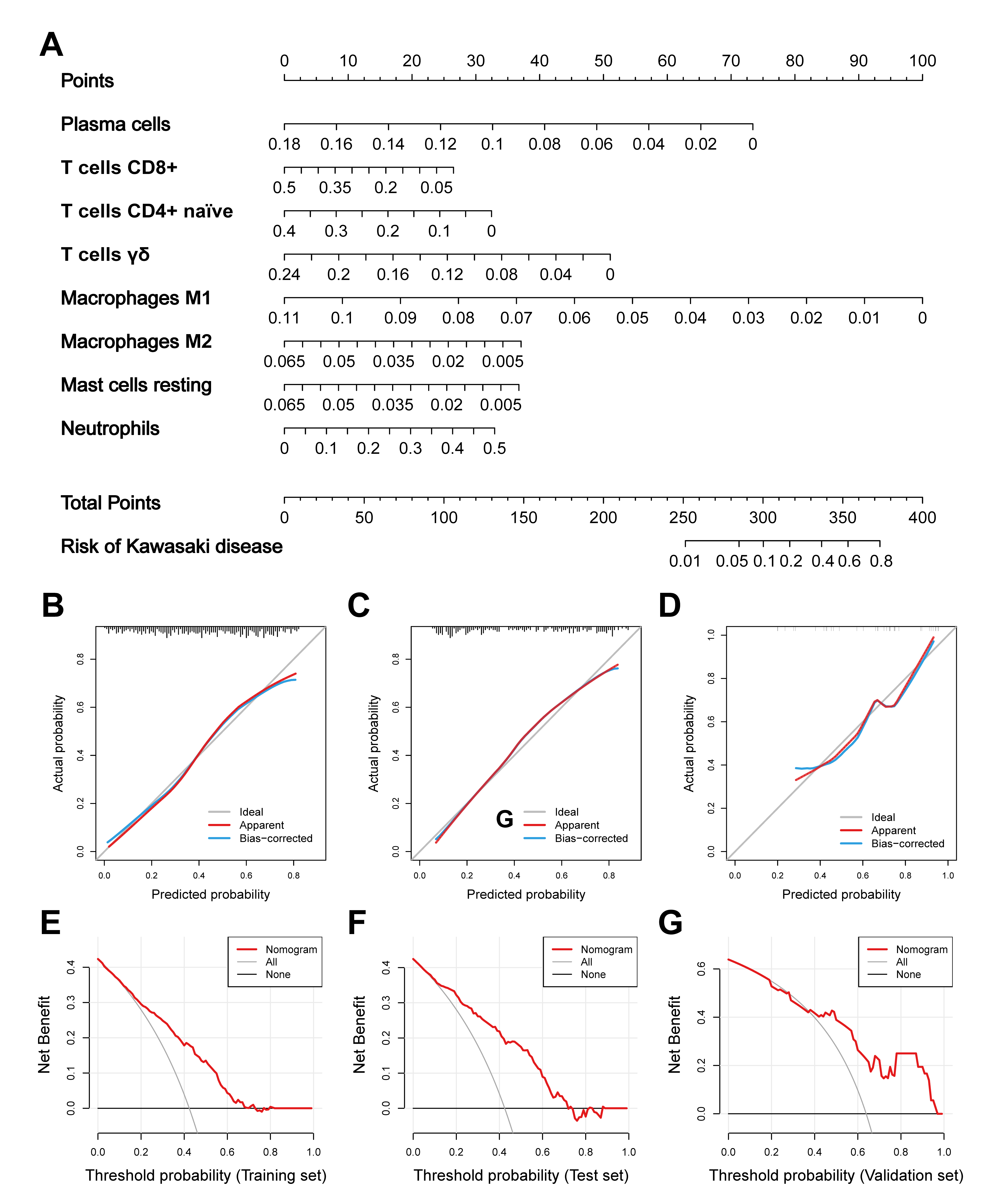

Supplement: Supplementary file 8 [file Image_3.TIF]

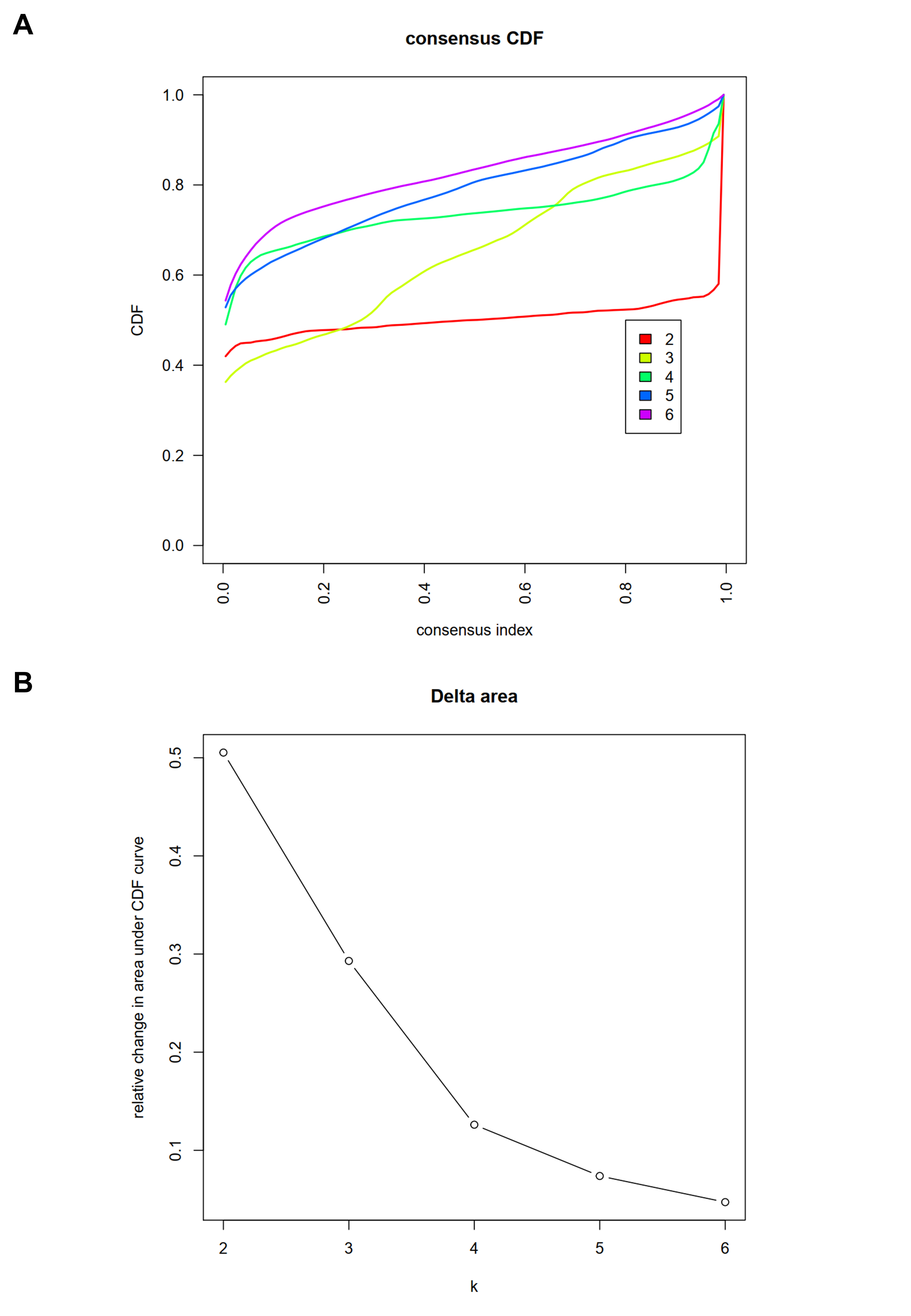

Supplement: Supplementary file 9 [file Image_4.TIF]
